# The Prevalence and Socio-Demographic Correlates of Depressive Symptoms in Early Adolescents in China: Differences in Only Child and Non-Only Child Groups

**DOI:** 10.3390/ijerph17020438

**Published:** 2020-01-09

**Authors:** Xinli Chi, Liuyue Huang, Jian Wang, Peichao Zhang

**Affiliations:** 1 College of Psychology, Shenzhen University, Shenzhen 518060, China; 1910481006@email.szu.edu.cn; 2 Shenzhen Key Laboratory of Affective and Social Cognitive Science, Shenzhen University, Shenzhen 518060, China; 3 College of Politics and Law, Anhui University of Architecture, Hefei 230000, China; wangj200434@163.com; 4 Research Center of Modern Psychology, Wuhan University, Wuhan 430071, China; patrickchang@foxmail.com

**Keywords:** depressive symptoms, early adolescents, gender, siblings, family functions

## Abstract

This study explores the prevalence and socio-demographic correlates of depressive symptoms in early adolescents in China, as well as the differences between an only child and non-only child group. A total of 2059 seventh-grade Chinese students were invited to complete a questionnaire, which included questions concerning socio-demographic factors, family function, and the Center for Epidemiological Studies Depression Scale (CES-D). The results revealed the following things. (1) thirty-four point seven percent of the participating Chinese early adolescents display symptoms of depression according to Radloff’s criteria. Differences are significant across the four dimensions (i.e., positive affect, negative affect, somatic symptoms and retarded activity, and interpersonal difficulties), as well as across total scores between only children and children with sibling(s). (2) Academic achievement, having sibling(s) or not, migration, and family function can significantly predict depressive symptoms. (3) Two significant interactions were found, which were between sibling(s) and gender as well as sibling(s) and family function. Girls from the non-only child group and adolescents from the only child group with poor family function were more likely to have depressive symptoms. These findings suggest that a greater focus should be placed on girls from non-only child families, academic under-performers, migrants, and adolescents from poor family environments, and especially only children, to prevent or reduce the propensity for depressive symptoms.

## 1. Introduction

Depression is an affective disorder with typical symptoms such as a persistent feeling of sadness, loss of sleep, lack of interest, and even suicidal thoughts which can damage the ability of daily functioning [1,2]. It has been a serious issue among adolescents and has been a leading cause of disability in adolescents globally [3]. In China, according to previous reports, the detection rate of self-rated depression in middle school students has been between 10% and 50% [4,5]. Several factors including personal (e.g., biochemistry, genetics, gender, and personality) and familial and social factors (e.g., family functioning and socioeconomic changes) might have an influence on depression [6,7]. The Health China Action for 2019–2030 has demonstrated the urgent need for a collective evidence-based effort to improve adolescent depression [8]. General interventions based on research evidence for depression include psychotherapy (e.g., family therapy and cognitive behavior therapy), pharmacotherapy, and a combination of the two [9,10]. Reducing depression calls for the joint efforts of government, society, researchers, and mental health professionals. Within this framework, this study aimed to assess the prevalence of depressive symptoms in early adolescents in China, along with socio-demographic correlates based on random large samples, to recognize risk factors and provide recommendations for the early detection of, prevention of, and/or clinical intervention for depression during adolescence.

The prevalence of depressive symptoms among adolescents varies greatly between different countries and regions. A study based on 11 European countries (e.g., France, Germany, and Italy) found that the rate of depressive symptoms in adolescents ranged 7.1% to 19.4% [11]. In Asia, according to a school-based study in North India, the prevalence of depressive symptoms amongst adolescents has reached 40% [12]. A survey conducted in Taiwan found that 30.2% of 9070 adolescents experienced depressive symptoms [13]. As for mainland China, researchers have found that Chinese teenagers are also at a high risk of developing depressive symptoms. For example, Zhou et al. [14] have found depressive symptoms ranging 14% in urban areas to 23% in rural areas. 

While many studies exist that focus on the prevalence of depressive symptoms among adolescents, there are several aspects that deserve further research. Firstly, previous research has covered a broad grade-range of adolescents, from primary school to high school, with little attention paid to early adolescents. Early adolescence is a developmental period during which individuals face important biological and social transitions, such as pubertal development, differentiation from parents, and creating new friendships (e.g., transitioning from primary school to middle school) [15]. These rapid changes mean that early adolescents are particularly vulnerable to mental health problems, such as depression. Previous studies have shown that the detection rate of depressive symptoms among early adolescents has been high, and that being depressed in early adolescence can lead to further negative outcomes in adult years, such as the development of asthma, diabetes, and epilepsy [16,17]. Thus, it is of great importance to carefully assess the prevalence and predictors of early adolescent depression. Secondly, some prevalence studies have been based on small samples (e.g., samples from one or two schools) or convenience sampling, which may lower the credibility of the derived results. Thus, the present study draws on a widely-used measure—the Chinese version of the Center for Epidemiologic Studies Depression Scale (CES-D)—to study depressive symptoms based on a large random sample of Chinese early adolescents.

The factors that relate to depression are also a focus of this research. According to ecological systems theory [18], individuals exist within a series of interdependent systems which influence them at different levels. The individual, as defined by their gender, age, and health, is the center of the system, and family is an important component of the microsystem (i.e., the direct environment the individual is in contact with) and the first point of socialization. As such, personal and familial factors may influence the growth of an individual. Moreover, Rutter and Garmezy [19] have proposed the developmental psychopathology theory (DPT) as an attempt to explain psychopathology problems (e.g., depression) of children and adolescents from the perspective of development. This perspective places abnormal development in a dynamic relationship between an individual and the situation in which he or she lives [20,21,22]. By understanding the individual, their environment, and the interaction between the two, we can better understand the developmental changes that occur in children and adolescents. Based on the aforementioned theories, we propose that personal factors, familial factors, and the interactions between these factors are related to levels of depression in Chinese early adolescents.

Regarding personal factors, studies have shown that depression in young adolescents rises significantly after 12 years of age [23]. The prevalence of depression in female adolescents has been shown to be significantly higher than that in males [24,25]. However, some literature has reported inconsistent results. For example, Bennett [26] has found the prevalence and severity of depression among boys and girls to be similar. These inconsistencies deserve further research. A correlation has also been found between depression in adolescents and the migration experience [27]. China has experienced rapid economic development and accelerated urbanization in the past several decades, which has led to a large number of migrant workers relocating to major cities. Previous studies have found that the mental health status of migrant adolescents is generally poorer than those of local adolescents in China [28]. For instance, one study covering 3254 participants in China found that the prevalence of depression in migrant adolescents was nearly twice that of local adolescents [29]. In addition, several studies have also suggested that an adolescent’s academic performance can predict their likelihood of developing depression [30]. The severity of depression in a group that achieved high academic results was found to be significantly less than that of groups that achieved average and poor academic results [30]. In the present study, it is assumed that older, female, migrant children, and children with poor academic performance, are more likely to experience depression (Hypothesis 1).

At the familial level, several factors have been shown to correlate with depression. For example, a relationship has been shown to exist between the education level of parents and youth depression. The lower the educational level of the parents, the higher the level of depression experienced by their children [31,32]. Adolescents from divorced families have been observed to be more prone to psychological problems such as depression [33]. Furthermore, family function is an essential variable associated with depression among adolescents [34]. Prior research has demonstrated that high family disharmony and parent–child conflict are significantly related to adolescent mental health problems (e.g., depression and suicidal ideation) [35]. Based on existing studies, this study assumed that low parental education, family non-intactness, and unhealthy family function are related to higher levels of depression in early adolescents (Hypothesis 2).

It is worth noting that even though China’s one-child policy ended in 2015, it was in place for over three decades and was mainstream in Chinese families [36]. The policy has attracted the attention of many researchers with regard to the psychosocial differences between groups of only children versus children with sibling(s). The resource dilution model posits that an increase in the number of children within a family leads to a reduction in available resources from parent to child [37,38]. In other words, family resources are limited, so as the number of children in the family increases, the less family resources each child can share [39]. Thus, non-only child families have to allocate limited family resources among multiple children, meaning the resources for each child are inevitably diluted. Empirical studies have demonstrated that having siblings may be a factor correlated with depression. For instance, a study covering 3397 students in Hefei, Anhui province, showed that the prevalence of depressive symptoms was 37.14% in one-child groups and 42.2% in not-one-child groups [40]. Moreover, a lack of sibling(s) may lead to changes in interactions between the subsystems of a family system, and thus may have different predictive effects on depression in an only child versus a non-only child group [41]. For instance, Zhang et al.’s [40] study showed that, compared to a group of children with sibling(s), academic stress was a significant predictor of depression in only children, and family intactness positively predicted depression in boys from non-only child families. Based on the literature, we speculated that the prevalence of depression is higher in non-only child groups compared to only child groups, and that there may be differences in correlates of depression between the two groups (Hypothesis 3).

Based on the background of previous research, the current study had three purposes. The first aim of the study was to investigate the prevalence of depression among Chinese early adolescents and to find out whether there was any difference in depressive symptoms between only child and non-only child groups. The second purpose of the study was to explore personal predictive factors (i.e., gender, age, having sibling(s) or not, and academic achievement) and family predictive factors (i.e., parentsal education level, family structure, and family function) of depression in Chinese early adolescents. The third aim was to further explore whether there are differences in predictors between only child and non-only child groups.

## 2. Methods

### 2.1. Participants

Data were collected from public schools between October and November, 2016, in Shenzhen, China. There are more than 150 public junior high schools in Shenzhen, among which 10 were randomly selected to participate in the study. The screening criteria for public middle schools in Shenzhen, China was having a local registered residence—known as “Hukou”—or having had a residence permit for over one year [42]. This work was a part of a three-year longitudinal project from the first year (Grade 7) to the third year (Grade 9) of junior high school. The first year of junior high school is a period of transition from primary school to middle school, which may bring many about adaptability problems (e.g., new teachers and peers, as well as greater study intensity and pressure compared to primary school) [43], so it is particularly meaningful to assign importance to this group. Thus, we chose Grade 7 students in early adolescence as our research participants. Before data were collected, informed consent forms were sent out to participants and their parents. Approximately 2342 Chinese seventh-grade students agreed to participate in the study. In total, 2059 students (87.9%) completed the questionnaire. The sample consisted of 1112 boys (54.0%) and 944 girls (45.8%) with a mean age of 12.44 years (SD = 0.63). Further demographic information concerning the participants is summarized in Table 1.

### 2.2. Procedure

This study was conducted by two psychology graduate students and a psychology teacher from each of the participating schools. Relevant training was conducted prior to the test, with standardized instructions used to ensure that each tester fully understood the requirements of the survey. Participants who completed the informed consent forms were divided into groups within their classrooms, with the participants sitting separately without any discussion with others. The test took around 25 min, and the questionnaires were collected on-site upon their completion. Testers were present throughout the questionnaire’s administration to answer any possible questions from participants. Students were encouraged to answer the questions honestly and were repeatedly assured of the confidentiality of the information that they were providing. The study and data collection were approved by the Human Research Ethics Committee of Shenzhen University.

## 3. Measurements

### 3.1. Depression

Depression was assessed using the Chinese version of the Center for Epidemiologic Studies Depression Scale. CES-D has been widely used in Chinese adolescents, with good reliability and validity [14,44]. CES-D consists of 20 items, each of which being answered on a four-point scale (0 = rarely or none of the time, 1 = some or a little of the time, 2 = moderately or much of the time, and 3 = most or all of the time), including four dimensions: negative affect (8 items), positive affect (4 items), somatic symptoms and retarded activity (6 items), and interpersonal difficulties (2 items). Positive affect was processed by reverse scoring, that is, the numerical scoring was run in the opposite direction, which was designed to improve the validity of the scale, and the larger value of this subscale stood for a less positive affect. The value of these 20 items was summed up, with the total value ranging from 0 to 60, where higher scores indicated a more severe level of depression. The scores of the four subscales were also summed up to compare the differences that may exist between only child and non-only child groups. A score of 15 points or less indicates no depression, while 16 points and above indicates depressive symptoms [45]. Cronbach’s α coefficient for this scale was 0.81.

### 3.2. Family Function

To assess participants’ family function, we used the family function scale for Chinese adolescents (C-FAI). The scale includes three subscales and a total of nine items, with each subscale including three items. Subscales include conflicts (e.g., “We have a lot of friction”), mutuality (e.g., “My family lives in harmony”), and communication (e.g., “In general, parents and children often have conversations”) [46]. All use a five-point Likert scale (1 = very dissimilar and 5 = very similar). After the conflict subscale reversed scoring was used, with the higher the sum of total score, the better the family function. Cronbach’s α coefficient for this study was 0.86, suggesting that this scale has good internal consistency reliability.

### 3.3. Social–Demographic Correlates

Participants were asked to report their gender (1 = male and 2 = female), age, whether they were only children (1 = yes and 2 = no), and their academic performance (1 = excellent, 2 = over average, 3 = average, 4 = below average and 5 = poor). They were also required to report their family status, including the level of parental education (1 = junior high school or below, 2 = high school, 3 = undergraduate, and 4 = above undergraduate), and family intactness (1 = integrity and 2 = divorced). All social–demographic data about the participants were counted in frequency and percentage, as summarized in Table 1. 

## 4. Statistical Analysis

Firstly, we calculated the frequencies and percentages of gender, age, one or more children, academic achievement, parents’ education level, family structure, and depressive symptoms. Next, an independent sample *t*-test was processed to examined the differences in the scores of CES-D and its four subscales between only child and non-only child groups, and we used Cohen’s *d* to measure their effect size [47]. Thirdly, a correlation analysis was carried out to investigate correlation between the variables. Fourthly, all continuous variables were centralized and a hierarchical multivariate regression analysis was used to examine the predictive effects of personal factors and familial factors on depression. Depression was regarded as a dependent variable and individual variables (gender, age, having a sibling or not, migration or no migration, and academic achievement) were considered independent variables, and were positioned in the first step. Family variables (parents’ education level, family structure, and family function) were considered independent variables, and were then placed in the second step; thirdly, interactions between sibling(s) and all the independent variables as independent variables were included in the analysis to assess any potential differences that may exist when a participant has sibling(s) versus having no siblings. If there were interactions, we further disentangled the variables that might about bring different effects. Categorical variables were dummy-coded as 0 (i.e., boy, migrant) or 1 (i.e., girl, local residence). All data were statistically analyzed using the Statistical Package for the Social Sciences (SPSS) version 23.0. When interpreting the results, statistical significance was set as *p* < 0.05 (two-tailed).

## 5. Results

### 5.1. The Prevalence of Depression among Early Adolescents

Thirty-four point seven percent of sample adolescents displayed depressive symptoms. As shown in Table 2, differences between the total score and the four dimensions of the CES-D between only child and non-only child groups were all statistically significant. The results were *t* = −4.94, *p* < 0.001 for total score; *t* = −3.09, *p* < 0.01 for negative affect; *t* = −4.93, *p* < 0.001 for positive affect; *t* = −3.51, *p* < 0.001 for somatic symptoms and retarded activity; and *t* = 2.76, *p* < 0.01 for interpersonal difficulties. Adolescents from non-only child families reported higher scores in negative affect, severe somatic symptoms, retarded activity, and interpersonal difficulties, with higher scores suggesting more severe symptoms, and had less positive affect. This indicated that adolescents from non-only child families exhibited more depressive symptoms than those from only child families. 

### 5.2. Correlation Analyses of the Variables

As shown in Table 3, we assessed five personal factors (i.e., gender, age, being from an only child or non-only child family, migration, and academic achievement) and four familial factors (i.e., education of the father, education of the mother, family intactness, and family function), which have been identified as relevant predictors of adolescent depression in previous research. Eight of the nine variables were observed to be significantly correlated with more severe depression (*r* between −0.07 and 0.41 and all *p* < 0.05), namely, older age, being from a rural area, having no sibling(s), being new migrants, poor academic achievement, and lacking family intactness. Gender was a marginally significant factor for depression (*r* = 0.04, *p* = 0.09).

A hierarchical regression analysis was applied to examine the predictive effects of personal and familial factors on depression. As shown in Table 4, in the first block, several socio-demographic variables were significantly associated with depression, and the model remained significant (*p* < 0.001). Gender was correlated with depression, and females were more likely to have depression (*β* = 0.04, *p* < 0.05); having siblings or not (*β* = 0.08, *p* < 0.001) showed a significant association, with adolescents from non-only child families at a higher risk of depression; and migrant status (*β* = −0.07, *p* < 0.001) was negatively correlated, indicating that migrants are more likely to have depression. Academic achievement (*β* = 0.21, *p* < 0.001) of participants could significantly predict depression, implying that students with poorer grades were more likely to have depression. However, age (*β* = 0.02, *p* > 0.05) did not produce a significant correlation. The second regression block included familial factors. Family function significantly predicted the tendency for depression (*β* = −0.37, *p* < 0.001), meaning that students from low-function families were more likely to have depression. However, parents’ education level (*β_fathe_*_r_ = 0.04, *β_mothe_*_r_ = −0.04, *p* > 0.05) and family intactness (*β* = 0.01, *p* > 0.05) did not show a significant correlation.

Two significant interactions in the third regression block were found between siblings and gender (*β* = 0.22, *p* < 0.05) and siblings and family function (*β* = 0.16, *p* < 0.05). In order to disentangle these interactions, regression analyses were re-run separately for the only child group and the non-only child group, without the main effects or interactions. The results of these analyses showed that for the only child group, gender was not related to depression (*β* = −0.009, *p* > 0.05). This was not the case for the non-only child group (*β* = 0.147, *p* < 0.05). In other words, girls in non-only child families are more likely to suffer from depression. Family function can negatively predict depression in both only and non-only child groups, but the standardized *β* value of the only child group (*β* = −0.435, *p* < 0.001) was more negative than in the non-only child group (*β* = −0.326, *p* < 0.001), indicating that for the only child group, family function had a stronger correlation to depression when compared to the non-only child group.

## 6. Discussion

This study has analyzed the prevalence and socio-demographic correlates of depressive symptoms in early adolescents in China and has looked at differences between an only child and non-only child group. Several findings have been interpreted as follows. Firstly, according to Radloff’s criteria [45], 34.7% of adolescents in our study displayed symptoms of depression, which indicates a generally high prevalence of depressive symptoms among Chinese early adolescents. This finding is similar to those in other areas of Asia, such as in Taiwan (30.2%) [13], and Korea (31.4%) [48]. However, the prevalence is higher than what Zhou et al. (2018) found in a national adolescent sample (20.3%) in China. Possible reasons for this might be that our study focused on early adolescents in the seventh grade who were within a transition period with a relatively high likelihood of depression [49]. In addition, our participants were from Shenzhen, a southeast coastal developed city of China, and due to the fast pace of modern cities and great academic competition and pressure, adolescents could be more prone to experiencing depressive symptoms [50]. Depression can lead to suicidal ideation and can induce self-injury and suicide [51]. Hence, there is a great need for researchers, educators, parents, and policy makers to work together to solve this issue. We also found that early adolescents in the non-only child group showed a greater negative affect, somatic symptoms, interpersonal difficulties, and a less positive affect, indicating that the general mental health status of non-only children was worse than that of only children, which is in line with previous studies conducted in China [52,53,54]. 

As predicted, the study found that having sibling(s) is a strong predictor of adolescents developing depression, which is consistent with previous studies [40,55]. Based on the resource dilution model, in families of only children, interpersonal resources such as attention, time, and energy provided by parents of only children may lead to better parental guidance and individual care. Thus, only children may adjust better psychologically and behaviorally [56]. Moreover, the only child group in China is embedded in a unique social, economic, and cultural background that has historical causes. According to Huang’s study [52], only children in China who generally live in areas with a more developed economy (e.g., Chinese registered residences—known as “Hukou”—in rural areas were legally permitted to have a second child if their first one was female), have parents with a higher level of education, and therefore a better occupational background (e.g., are staff in state-owned enterprises, government departments, and institutions), and have a richer and more diverse extracurricular life and thus a more pleasant childhood. These factors serve to protect their mental health, thereby reducing the likelihood of depression. 

Gender was another significant predictor, and early adolescent girls were more depressed than boys, in alignment with previous studies [57]. Two possible reasons for this could be that girls tend to have more ruminating thoughts than boys, which might play a role in the development of depressive symptoms, and also that girls are generally more vulnerable to stress than boys [58]. We also found that migration could significantly predict depression, which is consistent with prior findings [59,60]. The first and most commonly suggested reason for this is that the migration process causes stress, as migration entails an extensive loss of family and friends, customs, and surroundings [61]. In addition, migrants must adapt to a new cultural environment, which often includes different moral values and standards and a new language [62]. Certain adolescents who have undergone migration have psycho-social adaptation problems [63].

This study also found that academic achievement significantly predicted depression, showing that students with poor grades were more likely to have depression. Existing studies have also reached this conclusion [64,65,66]. It might be that students who perform poorly academically may be under pressure from their school and might receive negative feedback from parents and teachers, thus leading to low self-esteem [67]. Academic performance traditionally plays an important role in Chinese culture and is also one of the most important pursuits for contemporary Chinese teenagers [68]. The main activity of middle school students is learning and the measurement of achievement is also mainly aimed at the academic level. Stress comes not only from their own needs but also from teachers, parents, and peers. Adolescents mainly evaluate their abilities and their experiences of success or failure based on academic achievement [30]. Poor academic achievement may lead to shame and criticism, which may therefore increase the risk of depressive symptoms. In contrast, students who perform well tend to have higher self-esteem and receive positive feedback from peers, parents, and teachers, lowering their risk of depression.

Among the familial factors, as predicted, there was a significant correlation between family function and depression among adolescents. Poor family function predicted higher levels of depression. A comparative study between adolescents with emotional disorders and normal adolescents has found that adolescents with poor family function were more likely to develop psychological disorders such as depression, phobia, and anxiety [69]. Healthy family functioning positively predicts positive emotions in adolescents [70]. In contrast, teens in dysfunctional family settings receive more negative feedback and discouragement of emotional expression [71], leading to a low opinion of oneself and low self-esteem, which might cause mental problems such as depression.

Two significant interactions were found between siblings and gender and siblings and family function. Firstly, for the only child group, gender was not related to depression. However, for the non-only child group, girls were more likely to suffer from depression. This may be because females in only child groups are treated more equally, without any competition with sibling(s) for resources. Girls in non-only child families face a unique situation in China. Due to the concept of lineage, many people in China still have a son preference [72], so an illegal second, third, or even fourth child during the time of the one child policy may have been due to the parents’ desire to have a son. As such, one can infer that the developmental resources available to female children in such an environment may not be sufficient. The degree of family attention available to girls from non-only child families may be much lower than the attention available to girls who are only children. Thus, due to the fact that family and social care might not be enough for girls from non-only child groups, their probability of depression is likely to be increased [40].

Secondly, family function was a stronger predictor of depression in the only child group, indicating that only child adolescents are more likely to have depression if their family function is poor compared with non-only children. One possible reason for this is that if individuals live in a negative environment (e.g., with a bad parental relationship or parent–child relationship), having a sibling(s) may play a moderating role (i.e., a buffering effect) [73]. Looking at the structure of the only child family, adolescents must solely bear the pressure from parent–child conflicts [74]. However, for non-only children, interaction between brothers and sisters can alleviate any negative impacts from parent–child conflict [75]. This finding indicates that parents should attach more importance to building a positive family atmosphere to create a favorable environment for the healthy growth of adolescents.

This study also has some limitations. Firstly, its cross-sectional design enabled the researchers to discover a correlation but not a causal association. For example, the researchers were unable to determine whether negative academic achievement or family functioning will increase one’s chance of depression, or whether depression can lead to a decrease in academic achievement or family functioning. Future research should collect longitudinal data tracking early adolescents to examine the causal relationship between depression and the factors identified in this study. Secondly, the self-reporting approach used in the study may also be a limitation. Due to the social desirability effect, participants may under-report their depressive symptoms. Future research should use other research methods, such as experimental methods, to better understand this situation. Thirdly, this study only selected students from Shenzhen public junior high schools. Because public junior high schools have a certain screening for admission, the results of the investigation should only be generalized with caution. Future research could include students from private schools and special education schools. 

Despite these limitations, this study has examined the prevalence of depression in early adolescents in China and the relationship between depression and various demographic and family factors based on a large pool of randomized data. The investigation identified some implications for adolescent depression. Firstly, the prevalence of depression among early adolescents in China is relatively high, which suggests that urgent preventive or intervention measures are needed. Secondly, early adolescents in China with siblings, with poor academic performance, who are migrants or who have poor family function, need special help to prevent or reduce the possibility of developing depression. Last but not least, greater importance should be placed on education around gender equality and the mental health of girls from non-only child families.

## Figures and Tables

**Table 1:** Socio-demographic characteristics of the study participants.

Variables	*N* (%)	Variables	*N* (%)
Gender		Migration	
Boy	1112 (54.0)	Migrant	366 (17.8)
Girl	944 (45.8)	Local residence	1681 (81.7)
Not mentioned	3 (0.2)	Not mentioned	11 (0.5)
Only child family		Family intactness	
Yes	812 (39.4)	Intactness	1940 (94.2)
No	1243 (60.4)	Non-intactness	119 (5.8)
Not mentioned	4 (0.2)	Father education	
Age		Junior high school or below	590 (28.7)
11	33 (1.6)	High school	766 (37.2)
12	1195 (58.0)	Undergraduate	462 (22.4)
13	723 (35.1)	Above undergraduate	241 (11.7)
14	88 (4.3)	Mother education	
15	10 (0.5)	Junior high school or below	725 (35.2)
Not mentioned	10 (0.5)	High school	722 (35.1)
Academic achievement		Undergraduate	460 (22.3)
Excellent	172 (8.4)	Above undergraduate	149 (7.2)
Over average	652 (31.7)		
Average	698 (33.9)		
Below average	441 (21.4)		
Poor	94 (4.6)		

**Table 2:** Test of the difference in the total score and dimensions of depression between only children and non-only children in the early adolescence group.

Dimension	Only Child (*n* = 812) (*M* ± *SD*)	Non-Only Child (*n* = 1242) (*M* ± *SD*)	*t*	Cohen’s *d*
Total score	12.6 ± 9.29	14.73 ± 9.65	−4.94 ***	−0.22
Negative affect	4.22 ± 4.55	4.89 ± 4.89	−3.09 **	−0.14
Positive affect	4.80 ± 3.63	5.60 ± 3.51	−4.93 ***	−0.22
Somatic symptoms and retarded activity	2.80 ± 2.93	3.29 ± 3.12	−3.51 ***	−0.16
Interpersonal difficulties	0.78 ± 1.32	0.96 ± 1.49	−2.76 **	−0.13

Note: ** *p* < 0.01; *** *p* < 0.001. Scores of items were summed up, with higher scores indicating a more severe level of symptoms. Positive affect was processed by reverse scoring and a larger value stands for a less positive affect. Cohen’s *d* represents effect size, indicating to what degree differences are practical and meaningful.

**Table 3:** Correlation analysis of variables in early adolescence.

Variables	1	2	3	4	5	6	7	8	9	10
1 Gender	1									
2 Age	−0.12 ***	1								
3 Siblings	0.06 *	0.15 ***	1							
4 Migrant	0.06 **	−0.16 ***	−0.08 ***	1						
5 Academic	−0.04	0.12 ***	0.11 ***	−0.01	1					
6 Father education	0.05 *	−0.23 ***	−0.31 ***	0.16 ***	−0.2 ***	1				
7 Mother education	0.04 *	−0.23 ***	−0.36 ***	0.14 ***	−0.17 ***	0.71 ***	1			
8 Family intactness	0.01	−0.02	−0.1 ***	−0.04	0.04	0.08 **	0.07 **	1		
9 Family Function	0.01	−0.02	−0.08 ***	0.07 **	−0.21 ***	0.1 ***	0.13 ***	−0.16 ***	1	
10 CES-D score	0.04	0.07 **	0.11 ***	−0.08 ***	0.22 ***	−0.08 ***	−0.11 ***	0.07 **	−0.41 ***	1

Note: * *p* < 0.05, ** *p* < 0.01, *** *p* < 0.001. Gender: 0 = boy, 1 = girl; only child or not: 0 = only child, 1 = not only child; migration: 0 = migrant, 1 = local residence; family intactness: 0 = intactness, 1 = non-intactness. Legend: CES-D, Center for Epidemiologic Studies Depression Scale.

**Table 4:** Multiple linear regression of various variables on depression.

Variables	B	SE	*β*	*t*	ΔR^2^
**Step 1 Individual Variables**					0.06 ***
Age	0.02	0.02	0.02	0.9	
Gender	0.09	0.04	0.04	2.03 *	
Sibling(s)	0.15	0.05	0.08	3.42 ***	
Migrant	−0.18	0.06	−0.07	−3.08 **	
Academic achievement	0.21	0.02	0.21	9.49 ***	
**Step 2 Familial Variables**					0.13 ***
Age	0.03	0.02	0.03	1.42	
Gender	0.09	0.04	0.04	2.20 *	
Sibling(s)	0.11	0.04	0.05	2.40 *	
Migrant	−0.12	0.05	−0.05	−2.27 *	
Academic achievement	0.13	0.02	0.13	6.34 ***	
Father education	0.04	0.03	0.04	1.42	
Mother education	−0.04	0.03	−0.04	−1.63	
Family intactness	0.01	0.02	0.01	0.38	
Family function	−0.36	0.02	−0.37	−17.35 ***	
**Step 3 Interactions between Sibling(s) and All Variables**					0.01 *
Age	0.04	0.08	0.04	0.54	
Gender	−0.22	0.14	−0.11	−1.57	
Sibling(s)	−0.5	0.23	−0.24	−2.19 *	
Migrant	0.12	0.2	0.04	0.58	
Academic achievement	0.08	0.07	0.08	1.08	
Father education	0.02	0.09	0.02	0.27	
Mother education	0.08	0.08	0.08	1.03	
Family intactness	−0.09	0.06	−0.09	−1.5	
Family function	−0.52	0.07	−0.52	−7.06 ***	
Sibling(s) × age	−0.01	0.05	−0.02	−0.19	
Sibling(s) × gender	0.19	0.08	0.22	2.28 *	
Sibling(s) × migrant	−0.06	0.05	−0.09	−1.22	
Sibling(s) × academic achievement	0.03	0.04	0.06	0.78	
Sibling(s) × father education	0.02	0.05	0.03	0.36	
Sibling(s) × mother education	−0.09	0.06	−0.13	−1.63	
Sibling(s) × family intactness	0.31	0.18	0.19	1.73	
Sibling(s) × family Function	0.1	0.04	0.16	2.21 *	

Note: * *p* < 0.05; ** *p* < 0.01; *** *p* < 0.001. Coding was the same as in Table 3 above. B: unstandardized coefficients; SE: standard error; *β*: standardized coefficients.
